# Antagonistic effectiveness of *Anacardium occidentale* leaf extract on lead-acetate exposure-induced hepatorenal toxicity in rats

**DOI:** 10.5620/eaht.2023028

**Published:** 2023-12-29

**Authors:** Aisha Aminu, Hauwa Onozasi Umar, Wusa Makena, Zakaria Alhaji Isa, Zainab Muhammad Goni, Onyinoyi Bethel Onimisi, Barka Ishaku

**Affiliations:** 1 Department of Human Anatomy, Kaduna State University, Kaduna State, Nigeria; 2 Department of Human Anatomy, Federal University Dutse, Jigawa State, Nigeria; 3 Department of Human Anatomy, University of Maiduguri, Maiduguri, Borno State, Nigeria; 4 Department of Human Anatomy, Usmanu Danfodiyo University, Sokoto, Sokoto State, Nigeria

**Keywords:** Lead acetate, *Anacardium occidentale*, Succimer, Oxidative stress, Liver, Kidney

## Abstract

Lead (Pb) poisoning is an environmental substance that accumulates in the hepato-renal tissue, which is hazardous to health, while *Anacardium occidentale* L. is a tropical herb used to treat oxidative stress and inflammatory diseases. The aim of this study was to investigate the antagonistic effect of *Anacardium occidentale* leaf extract on lead acetate exposure-induced hepatorenal toxicity in rats. Thirty-six adult Wistar rats were split into six equal groups (n = 6). Group I served as a control, and groups II and III were administered lead acetate (50 mg/kg) and *Anacardium occidentale* leaf extract (400 mg/kg), respectively, while rats in groups IV–VI were administered *Anacardium occidentale* (L) extract (200 mg/kg and 400 mg/kg) and 10 mg/kg of Succimer, respectively, and were then administered lead acetate (50 mg/kg). When compared to the group I, rats administered lead acetate showed an increase in hepatic enzymes, urea, creatinine, MDA, TNF-α, and IL-1β (p < 0.001) levels and decreased levels of SOD, CAT, and GSH, whereas *Anacardium occidentale* prevented the increase in hepatorenal function parameters, oxidative stress, and inflammatory markers (TNF-α and IL-1β) induced by lead acetate. Rats administered only lead acetate had a marked increase in hepatic Pb concentration, severe hepatic steatosis, and renal glomerulus degeneration. However, treatment with *Anacardium occidentale* extract and succimer decreases the Pb concentration, oxidative stress, and inflammation, and also reduces histological liver steatosis and glomerular cytoarchitecture deterioration in the kidney. The results of this study revealed that *Anacardium occidentale* extract protects against lead acetate-induced liver and kidney toxicity by decreasing oxidative stress and inflammation.

## Introduction

Lead acetate (PbAc) is a persistent nonessential metal that is tasteless, odorless, and colorless. It remains in the environment for a very long time and can be found in the environment and living organisms in hazardous quantities [1]. Exposure to PbAc can be caused by PbAc paint, PbAc-containing gasoline, tainted drinking water, battery manufacturing, and industrial pollutants [2]. Pb is absorbed, goes through hepatic conjugation, and is then transported to the kidneys, where it is eliminated in minor amounts via urine. However, bulk PbAc accumulates in different organs. Post-mortem analysis of human corpses has shown that the liver, which stores approximately one-third of the total amount of ingested Pb, is the principal PbAc repository [3, 4]. PbAc is a multi-organ toxin that damages important organs such as the brain, liver, kidney, and testis, resulting in various illnesses and malignant tumors [5,6]. In addition to the central nervous system, which is one of the most severely affected organs, Pb diffuses into soft tissues and affects the kidneys and liver [7].

The mechanism underlying PbAc-induced oxidative stress, which results in damage to membranes, DNA, and proteins, is an imbalance between the synthesis and removal of reactive oxygen species (ROS) in tissue and cellular components [8]. PbAc affects the antioxidant defense systems of cells by inducing oxidative stress in cells. PbAc has been reported to alter antioxidant activity by inhibiting functional SH groups in several enzymes, including catalase (CAT), glucose-6-phosphate dehydrogenase (G6PD), superoxide dismutase (SOD), and glutathione peroxidase (GPx) [9,10]. The increased production of reactive oxygen and nitrogen species is detrimental [11, 12]. Routine PbAc chelators include succimer, dimercaprol (BAL), dimercaptosuccinic acid (DMSA), penicillamine, and EDTA. Chelating agents, however, are frequently associated with negative effects. Succimer, penicillamine, and BAL cause gastrointestinal problems, whereas EDTA causes calcium shortage and consequent kidney injury [13]. Furthermore, they are unable to restore related adverse effects, such as toxicant redistribution, metal loss, and hepatic or renal dysfunction [14,15]. Natural organic products are increasingly being used to mitigate the damaging effects of heavy metals and other hazardous pollutants. Because oxidative stress is the primary mechanism of PbAc toxicity, natural compounds high in antioxidants can act as antidotes to PbAc poisoning and can be used in conjunction with standard PbAc chelators [16, 17].

*Anacardium occidentale* L. (*A. occidentale*) is native to Brazil, although it is now grown worldwide in many tropical nations. *A. occidentale* leaf extracts have been widely used as folk medicine for various ailments in Thailand, Cameroon, and Nigeria [18,19]. Secondary metabolites found in *A. occidentale* plants have significant antioxidant [20,21], anti-inflammatory [22], and antimicrobial effects [20]. *A. occidentale* leaf extracts contain a high concentration of antioxidant bioactive secondary metabolites, including flavonoids, tannins, anthocyanins, alkaloids, quercetin 3-O-glucoside, quercetin 3-(2- galloylglucoside), quercetin 3-arabinoside, quercitrin, -tocopherols, and salicylic acid [23,24]. However, no research has been conducted on the hepatorenal protective effects of *A. occidentale* leaf extract against PbAc-induced hepatorenal toxicity in rats. The current study aimed to investigate the histological and biochemical effects of an ethanolic leaf extract of *A. occidentale* on PbAc-induced liver damage in adult Wistar rats.

## Materials and Methods

### Chemicals and Reagents

The hepato-renal toxicant lead (II) acetate trihydrate (PbAc) (CH3CO2)23H2O was obtained from Sigma-Aldrich (St. Louis, Missouri, USA). Succimer (meso-2,3 dimercaptosuccinic acid) (Sigma-Aldrich, MO, USA) was used as a standard drug (chelating agent). Ketamine hydrochloride (PVT Ltd., India) was used as the anesthetic agent. We bought our formaldehyde, hydrogen peroxide, hematoxylin, and eosin from Sigma Chemical Co. in St. Louis, Missouri, United States. The other chemicals and reagents used were of high analytical grade: alanine aminotransferase (ALT), aspartate aminotransferase (AST), alkaline phosphatase (ALP), and biochemical kits (Randox test kits). Antioxidant enzyme activities (CAT, SOD, and GSH) and MDA levels in blood serum were evaluated using laboratory diagnostic kits (Biodiagnostic Co., Cairo, Egypt).

### Plant Material

*A. occidentale* leaves were obtained from a local farm in Maiduguri, Jere Local Government, Borno State, Nigeria. A taxonomist from the Department of Botany at the University of Maiduguri performed the taxonomic identification and authentication. The voucher specimen, 2142, was housed in the herbarium of the department. The samples were then dried and pulverized in a knife mill. The pulverized plant material was concentrated in ethanol P.A (Ethyl alcohol) for 72 h to obtain the ethanolic extract, and the supernatant was removed and preserved. This method was repeated thrice for each row. The solvent was distilled using a rotary evaporator (Rotavapor R-300, Buchi) at 60 °C under decreased pressure. To complete the evaporation of ethanol, the ethanolic extract was placed in sterile glass vials and heated to 40 °C [25]. The crude extracts were placed in bottles, coated with aluminum foil, and refrigerated at 4 °C. The extract was administered to the rats in the form of daily suspensions in distilled water.

### Animals and Experimental Ethics Protocol

All experimental methods were carried out in compliance with ethical standards and guidelines that are congruent with the ARRIVE Guidelines, as approved by University of Maiduguri Directorate of Academic Planning and Monitoring (Approval No: (UM/HA/UGR20.21-0740067). Thirty-six Wistar albino rats weighing 150–160 g were obtained from the Department of Pharmacognosy and Therapeutic Maiduguri for this investigation. The rats were acclimated for two weeks in wire polypropylene cages under the following environmental conditions before being subjected to the treatment: 25 ± 2 °C, artificial 12 h light/12 h dark cycle. Animals were fed a standard diet and had unlimited access to water.

### Experimental design

Thirty-six (36) rats were randomly divided into six groups, and each group has six (6) rats. Group 1 served as the control and received only solvent (distil water) used to dissolve PbAc. PbAc was administered to Group 2 at a dose of 50 mg/kg/day, which has been previously associated with organ damage. Group 3 was administered only the extract (400 mg/kg) and was shown to be safe at this level [26], whereas Groups 4 and 5 were administered varying amounts of *A. occidentale* leaf extracts (200 and 400 mg/kg/day, respectively) and PbAc. Succimer (10 mg/kg) and PbAc were administered to Group 5. All treatments were administered orally for 45 days. The PbAc dosage employed in this investigation was shown to produce tissue toxicity and oxidative damage in rats [27, 28]. The *A. occidentale* leaf extracts concentrations used in this study were based on previous studies [26]. All the animals in each group were weighed before and after the experiment.

### Biochemical parameter assessment

Ketamine Hydrochloride (Pfizer, USA) Injection was administered intraperitoneally at a dose of 75 mg/kg to sedate the rats. At the completion of the study, all rats were sacrificed. Following sacrifice, blood was drawn by heart puncture and collected in simple, sterile, centrifuged bottles to clot. The liver organs were removed, weighed, and homogenized for the oxidative stress parameter in 50 mmol/l Tris-HCl buffer (pH 7.4) before centrifugation at 5000 × g for 15 min for biochemical analysis. The supernatants were quickly frozen and preserved until use. Blood serum was prepared by centrifuging the samples separately and storing them at -20 °C until the amounts of serum/homogenate were needed. Serum was used to estimate AST, ALT, ALP, creatinine, urea, and uric acid levels, whereas the supernatants from the liver tissue were used to estimate SOD, CAT, GSH, and MDA levels.

### Serum biochemical analyses

Serum biomarkers of liver and kidney damage were assessed using commercially available diagnostic kits (Randox Laboratories Ltd., UK) and the assay procedures provided by the manufacturer. Modified Wright et al. [29] methods were used to evaluate ALP activities, whereas modified Reitman and Frankel [30] methods were used to determine ALT and AST activities. Total proteins were determined using Stroeve and Makarova's [31] procedures. Renal parameters were estimated using the following methods: creatinine [32], serum urea [33], and uric acid [34].

### Supernatant Biochemical assessment

Using liver tissue homogenates, biochemical antioxidant and lipid peroxidation characteristics were investigated, and kinetic measurements were carried out for 2 min at 25 °C using a UV1800 spectrophotometer. MDA content was determined using Tsika's technique [35] to determine the level of lipid peroxidation. The method described by Jollow et al. [36] was used to determine GSH levels in the samples. CAT activity was assessed using a modified version of the method described by Chia et al. [37]. The procedure outlined by Fridovich [38] was employed to assess SOD activity.

### Hepatic Lead Concentration

According to the procedure outlined by Szkoda and Zmudzki [39], liver samples (weighing 2–10 g) were ovendried overnight at 120 °C before being placed in a muffle furnace at a low temperature that was progressively raised to 450 °C (50 °C/h). The material was digested with nitric acid after cooling. The samples were then placed in a muffle furnace at 450 °C/h and allowed to cool down. One gram of dry ash was dissolved in one mL of 1 N HCl. The final solutions contained 0.2% nitric acid added to them. The actual hepatic lead concentrations, expressed as g/g wet tissue weight, were analysed at 283.3 nm using flame atomic absorption spectrophotometry (Perkin-Elmer, 3200; CT, USA).

### Determination of pro-inflammatory cytokines

TNF-α and IL-6 activities in serum were measured using ELISA assay kits (Fine Test, Fine Biotechnology) according to the manufacturer’s instructions. The ELISA test results for pro-inflammatory cytokine activity are represented as picograms per milliliter of serum.

### Haematoxylin and Eosin staining method

The H and E staining was performed using the technique described by Bancroft and Stevens [40]. The tissues were dewaxed twice for three (3) minutes each, using two changes of xylene. After hydration with progressively higher concentrations of alcohol (100%, 95%, 90%, and 70%) for three minutes each, the sections were stained for ten minutes with Harris hematoxylin and then rinsed in tap water to eliminate any remaining stain. The slides were then cleaned again in tap water after being briefly submerged in acidic alcohol for distinction. The slides were then counterstained with Eosin for three minutes before being blued with Scott's tap water for five minutes. The parts were cleaned in xylene and washed in tap water before dehydration in varying degrees of alcohol.

### Rhodizonate staining method for lead Acetate

Lead deposition in histological sections of the liver was evaluated using sodium rhodizonate staining [40]. After washing in distilled water, the tissue samples were soaked in rhodizonate solution for an hour. It was then thoroughly cleaned in distilled water once more before being counterstained for 1 min with 0.05% aqueous quick green in 0.2% acetic acid. It was then rinsed three more times with distilled water, dried in progressively stronger alcohol, and cleaned in xylene. Subsequently, the parts were placed in an artificial resin. A digital microscope camera (AmScope MD 900) was used to obtain photomicrographs of the tissues under a light microscope at a magnification of X250.

### Statistical analysis

The mean and standard error of the mean (SEM) were obtained after statistical analysis of the data. The data were analyzed using one-way ANOVA, and a post hoc analysis was performed using Tukey's multiple comparison test. GraphPad Prism Software, version 9 (La Jolla, CA, USA) was used to conduct statistical analyses. The difference was deemed significant at P < 0.05.

## Results

### Effects of *A. occidentale* on the body and organ weights of rats exposed to PbAc

The weight gain of rats treated with PbAc decreased significantly (P < 0.05) when compared to rats in the NC group and the rats treated with only *A. occidentale* (AOLE), whereas AOLE + PbAc-treated rats gained weight significantly when compared with rats in the PbAc group (Table 1). Furthermore, rats administered only PbAc showed a significant (P < 0.05) increase in both liver and kidney somatic indices when compared with the somatic index of both organs in the control group and all the treatment groups (AOLE-PbAc and SUC-PbAc).

### Effects of *A. occidentale* on liver function parameters of rats exposed to PbAc

**Figure 1.**
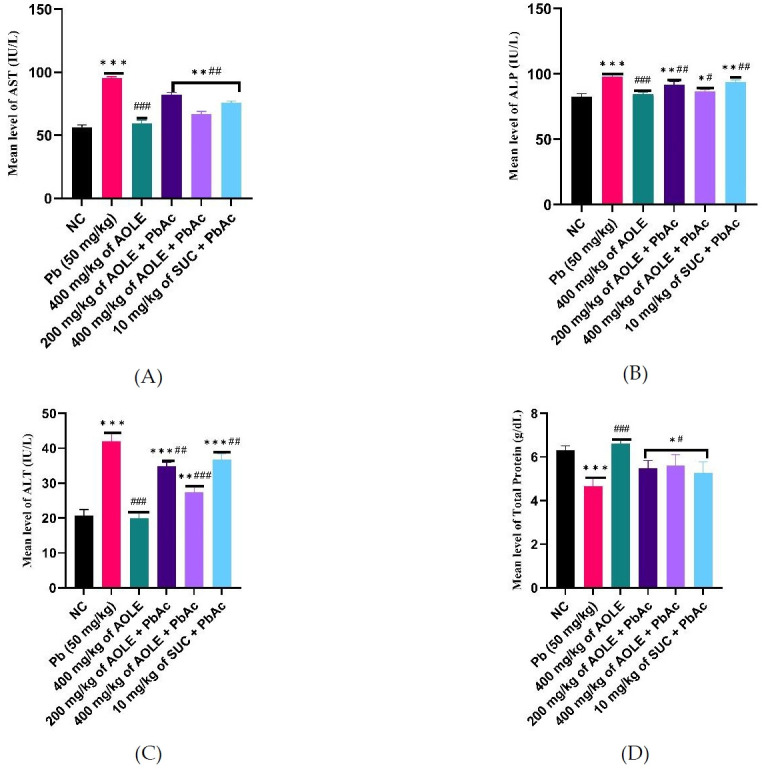
After 28 days of the experiment, a bar chart of the liver enzyme parameters (A) AST, (B) AST, (C) ALT, and (D) Total protein, ALP = alkaline phosphatase, ALT = alanine aminotransferase, AST = aspartate aminotransferase. #Significant difference compared with PbAc control #P<0.05; ##P<0.002; ###P<0.0001. *Significant difference compared with control *P<0.033; **P<0.002; ***P<0.0001. n=5

ALT, AST, and ALP levels in PbAc-treated rat serum were significantly higher (P < 0.05) compared to those in AOLE-PbAc and SUC-PbAc rats (Fig. 1). Consequently, treatment with AOLE-PbAc substantially and dose-dependently decreased liver enzymes compared to rats treated with PbAc. SUC-PbAc rats had considerably lower levels of ALP, AST, and ALT compared to PbAc rats (Figs. 1A, B, and C). The PbAc group had significantly (P < 0.001) lower blood total protein levels compared to the control group. At doses of 200 and 400 mg/kg, the total protein levels in the AOLE-treated rats were significantly higher (P < 0.05) than those in the PbAc group (Fig 1D).

### Changes in kidney function parameters and hepatic tissue PbAc concentration

**Figure 2.**
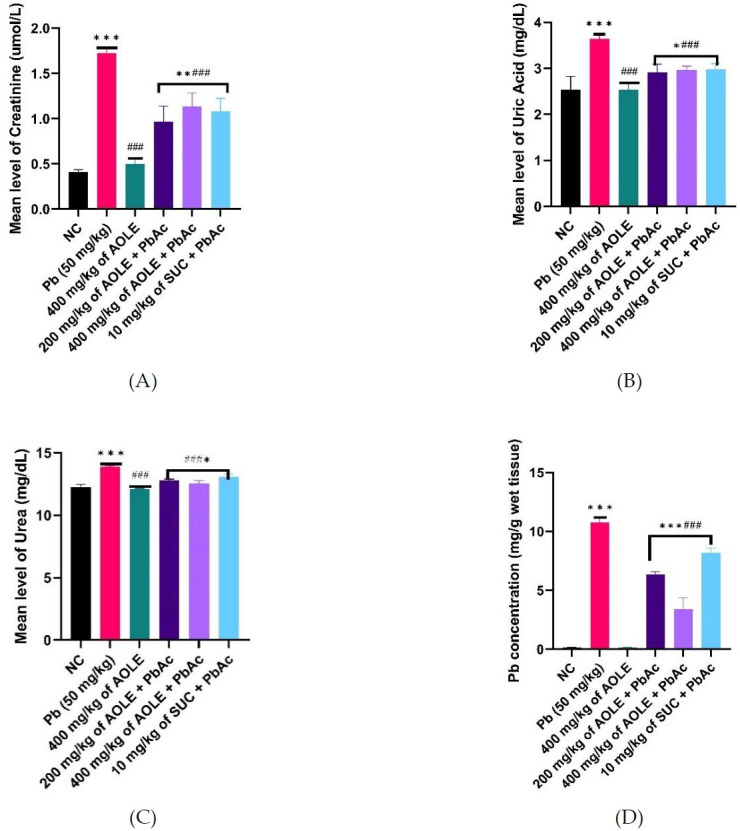
Bar charts of kidney function parameters (A) creatinine (B) Uric Acid, and (C) Urea and (D) Pb concentration after 28 days of the experiment. #Significant difference compared with PbAc control #P<0.05; ##P<0.002; ###P<0.0001. *Significant difference compared with control *P<0.033; **P<0.002; ***P<0.0001. n=5.

The PbAc groups showed significantly (P < 0.001) increased serum levels of uric acid, urea, and creatinine compared to the NC and AOLE-treated groups (Fig. 2 A, B & C). AOLEPbAc-treated rats showed significantly (P < 0.05) reduced levels of uric acid, urea, and creatinine at doses of 200 and 400 mg/kg compared to the PbAc group (P < 0.05). Urea, uric acid, and creatinine levels were also significantly higher in the AOLE-PbAc group than in the NC and AOLE-treated groups. The levels of renal biomarkers (urea, uric acid, and creatinine) in the SUC-PbAc group were significantly lower than those in the PbAc group. There was no significant difference (P > 0.05) in urea, uric acid, and creatinine levels between the AOLE-PbAc and SUC-PbAc groups. The mean hepatic Pb concentration in PbAc-treated rats increased significantly (p < 0.001) when compared to rats in the NC group. Compared to the PbAc-treated group, there was a significant decrease in hepatic PbAc concentration in the AOLE-PbAc and SUC-PbAc-treated groups. The hepatic tissue PbAc concentration showed no significant difference between the AOLE + PbAc-and SUC + PbAc-treated groups (P > 0.0%) (Fig. 2D).

### Effects of *A. occidentale* on the oxidative stress parameters of rats exposed to PbAc

**Figure 3.**
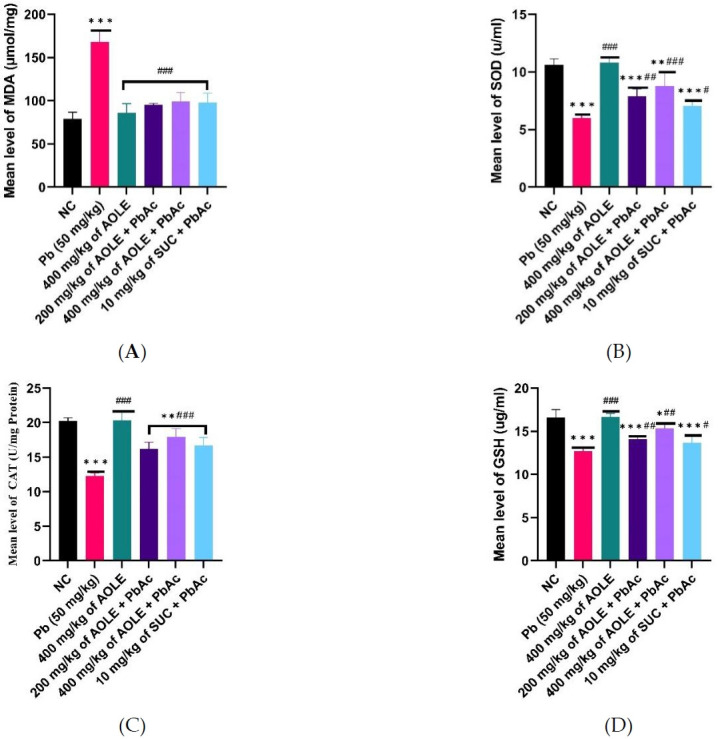
Bar charts of oxidative stress parameters ((A) MDA, (B) SOD, (C) CAT and (D) GSH) after 28 days of the experiment. GSH = glutathione reductase, CAT = catalase, MDA = malondialdehyde, SOD = superoxide dismutase #Significant difference compared with PbAc control #P<0.05; ##P<0.002; ###P<0.0001. *Significant difference compared with control *P<0.033; **P<0.002; ***P<0.0001. n=5.

Compared to the rats in the NC and AOLE groups, only PbAc-treated rats had substantially lower levels of GSH, SOD, and CAT enzyme activity. Additionally, SOD, CAT, and GSH levels were significantly increased (P < 0.05) in the AOLE and succimer-treated rats (200 mg/kg AOLE + PbAc, 400 mg/kg AOLE + PbAc, and 10 mg/kg SUC + PbAc) than in the PbActreated rats. Rats in the 400 mg/kg AOLE + PbAc group showed significantly higher levels of SOD, CAT, and GSH compared to those in the 200 mg/kg AOLE + PbAc and 10 mg/kg SUC + PbAc groups. The rats in the 200 mg/kg AOLE + PbAc and 5 mg/kg SUC + PbAc treatment groups did not vary significantly (P > 0.05) (Fig. 3A-C). MDA levels were significantly higher in the PbAc group compared to the NC group. Compared to the PbAc group, AOLE and SUC significantly (p < 0.05) reduced MDA levels (Fig. 3D).

### Effect of *A. occidentale* on TNF-α and IL-6 in ethanol-induced toxicity

**Figure 4.**
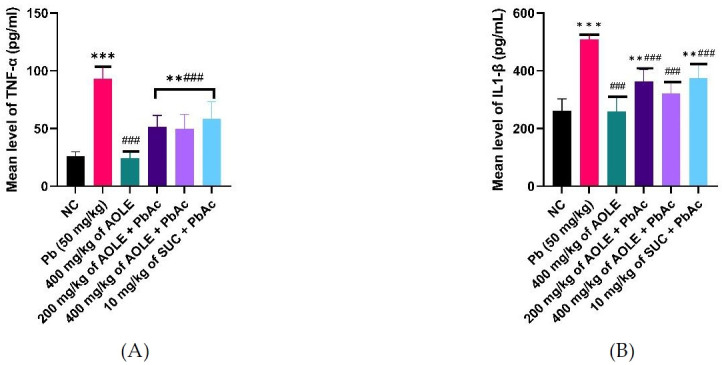
Bar charts of Inflammatory markers (A) TNF-α and (B) IL-6. TNF-α =, Tumour Necrosis Factor Alpha.; IL-1 β = interleukin 1β #Significant difference compared with PbAc control #P<0.05; ##P<0.002; ###P<0.0001. *Significant difference compared with control (NC) *P<0.033; **P<0.002; ***P<0.0001. n=5.

The mean TNF-α and IL-6 levels are shown in Figure 4. TNF-α levels were significantly higher (P < 0.05) in PbAc control rats (PbAc) than in normal control (NC) rats and rats treated only AOLE-treated (400 mg/kg AOLE). TNF-α and IL6 levels in the 200 mg/kg AOLE + PbAc and 400 mg/kg AOLE + PbAc groups were significantly lower (P < 0.05) than those in the PbAc control group. The levels of TNF-α and IL-6 in Succimer-treated rats (10 mg/kg SUC + PbAc) were not significantly different (P > 0.05) from those in the low-dose flavonoid-treated rats (Fig. 4A and 4B).

### Effects of *A. occidentale* on the histopathology of rats exposed to PbAc

**Figure 5.**
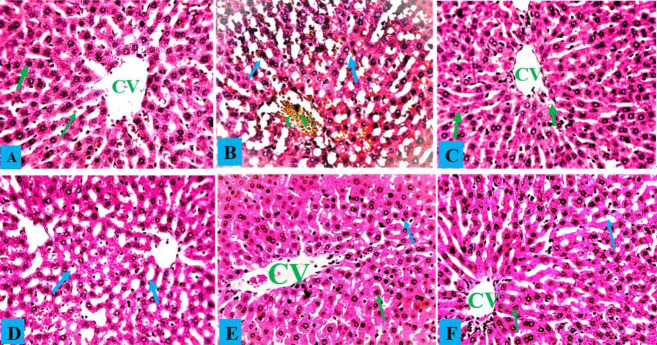
Control liver photomicrograph (A) and AOLE treated liver (C) showed with normal hepatocytes (green arrow) and central vein (CV); PbAc treated liver photomicrograph (B) with remarkable degenerating hepatocytes and fat hepatocellular vacuoles (blue arrow). AOLE 200/400mg/kg + 50 mg/kg PbAc (D&E) demonstrated mild inflamed hepatocyte (green arrow) and normal hepatocyte; Succimer + 50 mg/kg PbAc treated rats (F) demonstrated mild vacuolated hepatocyte (blue arrow). H and E staining at X200 magnification.

**Figure 6.**
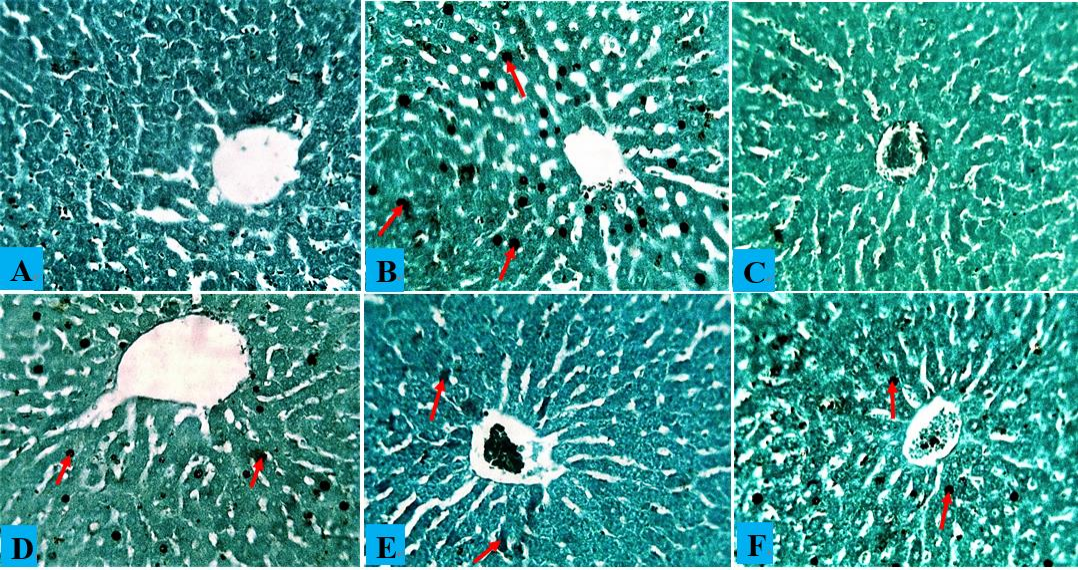
(A) Composite photomicrographs of control (A) and liver of rats treated with only AOLE (C), showing normal distribution cytoarchitecture of the liver; PbAc treated liver photomicrograph (B) showing excess accumulation of PbAc residue within the liver cytoarchitecture (blue arrow). AOLE 200/400mg/kg + 50 mg/kg PbAc (D&E) demonstrated mild accumulation of PbAc within the hepatocyte (blue arrow) in a dose dependent manner, the hight dose the PbAc concentration is very minimal; Succimer + 50 mg/kg PbAc treated rats (F) demonstrated mild deposit of the PbAc (red arrow). sodium rhodizonate staining at X200 magnification.

Hepatocytes, central veins, and sinusoids in the liver of a healthy control rat showed no abnormalities, and the same situation was observed in rats administered only AOLE (Fig. 5A and C). Rats treated with PbAc showed hepatocyte degeneration due to steatosis and fat hepatocellular vacuoles in the liver (Fig. 5B). The histology of rats treated with AOLE (200 mg/kg) and PbAc (50 mg/kg) showed mildly degenerated hepatocytes, some micro-vesicular fat droplets, and vacuolated hepatocytes (Fig. 5D), whereas the livers of rats treated with AOLE (400 mg/kg) and PbAc (50 mg/kg) showed normal hepatic cells, sinusoids, and central veins, with scanty vacuolated hepatocytes (Fig. 5E). Rats administered concurrent treatments with Succimer (10 mg/kg) and PbAc (50 mg/kg) had slightly vacuolated livers and deteriorated hepatocytes (Fig. 5F). In both the control and AOLE groups, rhodizonate staining for lead acetate in the liver revealed normal histoarchitecture of the liver parenchyma stained green and free from lead acetate accumulation (Fig. 5A and C). Lead acetate was markedly deposited in the liver parenchyma in liver sections from PbAc (50 mg/kg)-treated rats (Fig. 5b). Rat liver sections were co-administered AOLE (200 mg/kg) + PbAc and SUC (5 mg/kg) + PbAc. In both the control and AOLE groups, rhodizonate staining for lead acetate in the liver revealed normal histoarchitecture of the liver parenchyma stained green and free from lead acetate accumulation (Fig. 6A and C). Lead acetate was markedly deposited in the liver parenchyma in liver sections from PbAc (50 mg/kg)-treated rats (Fig. 6B). Rat liver sections were co-administered AOLE (200 mg/kg) + PbAc and SUC (5 mg/kg) + PbAc. In the liver parenchyma of the experimental rats, PbAc revealed a modest accumulation of lead acetate (Figs. 6D and F), but liver sections from the animals co-administered AOLE (400 mg/kg) and PbAc revealed very few PbAc deposits (Fig. 6E).

**Figure 7.**
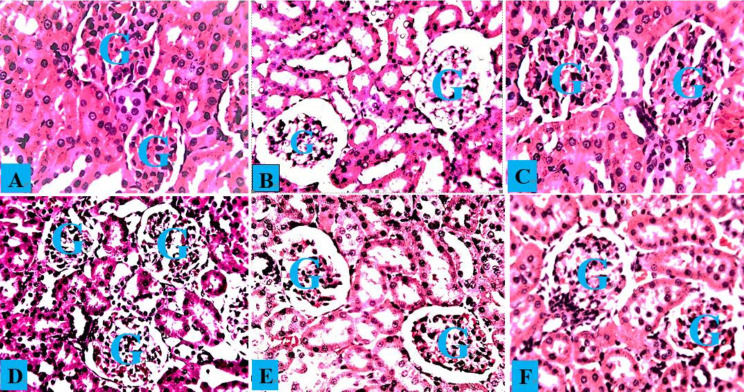
Composite photomicrographs of the kidney from the control group (A) and AOLE (C) show a normal glomerulus (G) and renal tubules; the PbAc-treated micrograph (B) shows spontaneous lipid vacuolation of the glomerulus (G); the AOLE 200mg/kg + 50 mg/kg PbAc micrograph (D) displays mildly degenerated and spontaneous lipid vacuolation of the glomerulus; the AOLE 400mg/kg + 50 mg/kg PbAc micrograph (E) showing very normal rental tubules and normal glomerulus (G), Succimer treated group (E), exhibited mild obliterative form of glomerulus (G). H and E staining at X200 magnification.

The kidney histology of normal control rats and rats treated with *A. occidentale* revealed a typical histological structure with normal glomeruli and renal tubules (Figs. 7A and 7C). The kidneys of PbAc-treated rats had significantly deteriorated glomeruli, spontaneous lipid vacuolation of the glomerulus, and localized renal tubular degeneration (Fig. 7B). Rats administered both AOLE (200)-PbAc and SUC (10 mg/kg)-PbAc had slight sinusoid dilatation and vacuolation of the glomerulus (Fig. 7D and F), while rats administered only AOLE (200)-PbAc had a normal glomerulus and some modest tubular injury (Fig. 7E).

## Discussion

PbAc toxicity is becoming a global concern, and researchers are paying close attention to it because of its numerous applications. It is a poisonous and nondegradable metal that cannot be altered by biological processes into harmless molecules [1, 41]. In recent studies, compounds of plant origin have been used to treat PbAc toxicity with little to no side effects. Notably, *A. occidentale* treatment offers defense against tissue damage caused by a variety of toxins owing to its antioxidant capabilities [26]. Hence, in this study, we evaluated the protective effects of two different dosages of *A. occidentale* on biochemical changes in the kidney and liver tissue, and the oxidative stress induced by PbAc in Wistar rats. In comparison to controls, rats administered PbAc (50 mg/kg) orally experienced a significant decrease in body weight gain (P < 0.05), according to the study's findings. The results of this study are consistent with previous findings, and they might be explained by increased catabolism and anorexia, which are linked to reduced food intake and weight loss induced by PbAc [42]. Furthermore, the loss of renal water reabsorption-related tubular cells results in dehydration and a decrease in body weight [43], whereas rats treated with both doses of *A. occidentale* were able to protect against these PbAc-related negative effects.

Following PbAc exposure, a significant increase in the levels of liver and kidney biomarkers (ALT, ALP, AST, urea, creatinine, and uric acid) was observed in this study, which is consistent with previous studies [16, 44]. The increase in these markers was caused by both the rupture of the glomerular filtration barrier and alterations in hepatocyte membrane permeability, which allowed these enzymes to seep into the circulation [45, 46]. High levels of key markers for liver and renal dysfunction have been used to support the hypothesis that Pb causes hepatorenal toxicity [47, 48]. Free radicals produced by PbAc, which increase levels of lipid peroxidation and compete with calcium, a crucial component for maintaining the integrity of cell membranes, may be responsible for the disruption of the hepatic membrane's permeability and the glomerular filtration membrane [45]. Treatment with *A. occidentale* significantly decreased the levels of biomarkers for liver and kidney function, suggesting that *A. occidentale* might protect the integrity of hepatocytes and kidneys, and protect both organs from injury. This was consistent with the observations of Olajide et al. [49] and Baptista et al. [26]. The hepatic necrosis and degeneration induced by PbAc exposure were shown to be attenuated by *A. occidentale* treatment by the histological screening of both tissues (Fig 3, 4 & 5).

The antioxidant enzymes SOD, GSH, and CAT were decreased, and the MDA level was significantly increased in the hepatic tissues of rats exposed to PbAc, which is in line with previous findings [41, 50]. The main mechanism by which PbAc induces hepatic stress is thought to be its ability to increase reactive oxygen species (ROS) production. ROS can increase lipid peroxidation and deplete the antioxidant reserves in cells. Additionally, several enzymatic and non-enzymatic antioxidant sulfhydryl groups have high binding affinities for PbAc, which reduces the effectiveness of such antioxidants [51, 52]. Some important ions, including zinc and copper, which serve as crucial cofactors in the activities of SOD and CAT, can compete with and replace PbAc [43]. Treatment with *A. occidentale* prevented PbAc-induced oxidative stress by elevating SOD, CAT, and GSH activities and lowering MDA levels in hepatic tissue. The antioxidant potential of *A. occidentale* has been reported previously [26]. Flavonoids, components of *A. occidentale*, might exert their antioxidant properties through several mechanisms involving the ability to trap ROS via chain-breaking properties, thereby exerting their defensive effects on cellular macromolecules, in addition to increasing the cellular level of total GSH and suppressing the peroxidation of lipids [53]. Additionally, *A. occidentale* employs its antioxidant ability by scavenging ROS and RNS, chelating transition metals such as iron and copper that may cause oxidative damage through the Fenton reaction, suppressing pro-oxidant enzymes, and enhancing the activities of antioxidant enzymes [54]. Anacardic acids (AAs) are a class of compounds found in Anacardium occidentale that have been linked to anti-inflammatory and antioxidant properties [55, 56]. These substances are most likely to aid in lowering ROS/RNS. Additionally, AAs efficiently inhibit pro-oxidant enzymes that produce ROS and RNS. Additionally, AAs have the chemical characteristics of chelating divalent metal ions and can thus inhibit processes such as the Fenton reaction, leading to the generation of ROS and RNS [57].

A pathogenic event associated with the liver and kidney process is the inflammatory reaction [58], which is associated with the overproduction of proinflammatory cytokines such as TNF-α and IL-1β [59, 60]. These cytokines play a vital role in the development of toxicity, as commonly demonstrated in animal models. The expression of inflammatory mediators and the generation of pro-inflammatory cytokines (including TNF-α and IL-1β) are facilitated by the activation of NF-kB in the hepatocellular nucleus, which in turn leads to inflammation and apoptosis and might produce ROS [61]. As a result, NF-kB is widely established as a key modulator of cellular necrosis and inflammation [62]. From these studies, it was found that PbAc treatment remarkably enhanced the levels of TNF-α and IL-1β in rat [4]; however, both groups of AOLE + PbAc administration inhibited these elevations, which was attributed to the significant reduction in the serum levels of cytokines (TNF-α and IL-1β) elevated by PbAc. The NF-kB pathway is likely used by these *A. occidentale* extracts to control the inflammatory response, with different effects on downstream proteins. This might be attributed to the flavonoid content in *A. occidentale*, as previous research indicated that flavonoids might lower the levels of inflammatory factors by inhibiting NF-kB [63, 64].

In this current study, the hepatic tissue of the PbAc-exposed group showed a substantial increase in PbAc residues, which was consistent with earlier findings [4, 65]. PbAc accumulates largely in the liver and kidneys, because these organs are the primary locations for Pb excretion and contain proteins with a high affinity for PbAc binding, such as metallothionein, acyl-CoA binding protein, and thymosin β4 [66]. In addition, the group treated with AOFE + PbAc showed a significant decrease in hepatic PbAc concentration. According to a recent study, *A. occidentale* may offer defense against heavy metalinduced toxicities owing to its chelating potential, which may be caused by its high flavonoid concentration [20, 65]. Metalflavonoid complexes can be formed by metals and flavonoids because they have the potential to chelate metals. The carbonyl and hydroxyl functional groups that are connected to the ring structures of flavonols give them the capacity to form metal complexes [67].

## Conclusions

This study found that PbAc (50 mg/kg) created an oxidative imbalance in the liver and renal tissues of rats, resulting in oxidative stress and pathological damage. By enhancing antioxidant defense mechanisms and lowering the levels of lipid peroxidation in tissues, oral treatment with *A. occidentale* at doses of 200 and 400 mg/kg/day significantly prevented PbAc-induced toxicity in the liver and kidney tissues. Thus, we conclude that *A. occidentale* might be regarded as a natural supplement with the potential for protection/prevention against PbAc overload in hepatocellular tissues.

## Figures and Tables

**Table 1.** Initial and final body weights as well as weight variations in the rat’s kidney and liver organ.

Groups	Initial wt (g)	Final wt. (g)	Body wt gain (g)	Liver Wt (g)	Kidney Wt (g)	Hepatic Somatic Index	Renal Somatic Index
NC	154.67 ± 1.94	196.83 ± 4.19	42.00 ± 2.57	5.30 ± 0.16	0.85 ± 0.029	2.69 ± 0.045	0.42 ± 0.007
PbAc (50 mg/kg)	156.17 ± 2.07	175.17 ± 3.10^*^	18.50 ± 1.06^***^	5.35 ± 0.05	0.84 ± 0.014	3.05 ± 0.060^*^	0.48 ± 0.011^*^
400 mg/kg AOLE	155.65 ± 1.43	197.00 ± 2.33^#^	41.33 ± 1.20^###^	5.15 ± 0.05	0.84 ± 0.019	2.62 ± 0.049^#^	0.42 ± 0.008^#^
200 mg/kg AOLE + PbAc	155.17 ± 2.26	180.83 ± 2.49^*^	25.67 ± 1.45^#**^	4.87 ± 0.22	0.81 ± 0.008	2.69 ± 0.049^#^	0.45 ± 0.004^#^
400 mg/kg AOLE + PbAc	156.50 ± 2.05	189.67 ± 2.35^#^	33.17 ± 0.95^##^*	5.13 ± 0.08	0.82 ± 0.026	2.71 ± 0.053^#^	0.42 ± 0.012^#^
10 mg/kg SUC + PbAc	153.83 ± 1.57	177.00 ± 1.51^*^	23.81 ± 1.14^#^**	4.97 ± 0.09	0.80 ± 0.016	2.80 ± 0.088^#^	0.45 ± 0.008^#^
F	0.268	12.495	42.589	1.889	1.544	4.226	4.622
p-Value	0.927	0.000	0.000	0.126	0.206	0.005	0.003

# Significant difference compared with PbAc control # P<0.05; ## P < 0.002; ### P < 0.0001.

* Significant difference compared with control * P<0.033; ** P < 0.002; *** P < 0.0001.

## List of Abbreviations

AAs: Anacardic acids
ALP: Alkaline phosphatase
ALT: Alanine aminotransferase
AO: Anacardium occidentale L.
AOLE: Anacardium occidentale leave extract
AST: Aspartate aminotransferase
CAT: Catalase
G6PD: Glucose-6-phosphate dehydrogenase
GPx: Glutathione peroxidase
GSH: Reduced glutathione
MDA: Malondialdehyde
NC: Normal Control
PbAc: Lead (II) acetate
RNS: Reactive nitrogen species
ROS: Reactive oxygen species
SOD: Superoxide dismutase
SUC: Succimer
